# Exogenous L-Glutathione Improves Vitrification Outcomes in Murine Preimplantation Embryos

**DOI:** 10.3390/antiox11112100

**Published:** 2022-10-25

**Authors:** Nor-Shahida Abdul Rahman, Nor-Ashikin Mohamed Noor Khan, Zolkapli Eshak, Mimi-Sophia Sarbandi, Aqila-Akmal Mohammad Kamal, Mastura Abd Malek, Fathiah Abdullah, Maizaton Atmadini Abdullah, Fezah Othman

**Affiliations:** 1 Maternofetal and Embryo Research Group (MatE), Faculty of Medicine, Universiti Teknologi MARA, Selangor Branch, Sungai Buloh Campus, Sungai Buloh 47000, Selangor, Malaysia; 2 Faculty of Medicine and Health Sciences, Universiti Putra Malaysia, Serdang 43400, Selangor, Malaysia; 3 Faculty of Pharmacy, Universiti Teknologi MARA, Selangor Branch, Puncak Alam Campus, Bandar Puncak Alam 42300, Selangor, Malaysia; 4 Faculty of Applied Sciences, Universiti Teknologi MARA, Perak Branch, Tapah Campus, Tapah Road 35400, Perak, Malaysia

**Keywords:** cryotolerance, embryo viability, preimplantation development, L-glutathione, vitrification

## Abstract

Vitrification is an important tool to store surplus embryos in assisted reproductive technology (ART). However, vitrification increases oxidative damage and results in decreased viability. Studies have reported that L-glutathione (GSH) supplementation improves the preimplantation development of murine embryos. Glutathione constitutes the major non-protein sulphydryl compound in mammalian cells, which confers protection against oxidative damage. However, the effect of GSH supplementation on embryonic vitrification outcomes has yet to be reported. This study aims to determine whether GSH supplementation in culture media improves in vitro culture and vitrification outcomes, as observed through embryo morphology and preimplantation development. Female BALB/c mice aged 6–8 weeks were superovulated through an intraperitoneal injection of 10 IU of pregnant mare serum gonadotrophin (PMSG), followed by 10 IU of human chorionic gonadotrophin (hCG) 48 h later. The mated mice were euthanized by cervical dislocation 48 h after hCG to harvest embryos. Two-cell embryos were randomly assigned to be cultured in either Group 1 (GSH-free medium), Group 2 (GSH-free medium with vitrification), Group 3 (0.01 mM GSH-supplemented medium), or Group 4 (0.01 mM GSH-supplemented medium with vitrification). Non-vitrified (Groups 1 and 3) and vitrified (Groups 2 and 4) embryos were observed for morphological quality and preimplantation development at 24, 48, 72, and 96 h. In the non-vitrified groups, there were significant increases in the number of Grade-1 blastocysts in GSH cultures (*p* < 0.05). Similarly, in the vitrified groups, GSH supplementation was also seen to significantly increase blastocyst formation. Exogenous GSH supplementation resulted in a significant increase in intracellular GSH, a release of cytochrome c from mitochondria, and a parallel decrease in intracellular reactive oxygen species (ROS) levels in vitrified eight-cell embryos (*p* < 0.05). GSH supplementation was shown to upregulate *Bcl2* expression and downregulate *Bax* expression in the vitrified preimplantation embryo group. The action of exogenous GSH was concomitant with an increase in the relative abundance of *Gpx1* and *Sod1*. In conclusion, our study demonstrated the novel use and practical applicability of GSH supplementation for improving embryonic cryotolerance via a decrease in ROS levels and the inhibition of apoptotic events by improvement in oxidative status.

## 1. Introduction

Vitrification involves short exposures to high concentrations of cryoprotectants, with extreme cooling and warming rates. It has the unique physical feature of preserving living cells in a glass-like state without the formation of ice crystals. The technique has been successfully applied to humans, as well as to several livestock species. Vitrification is preferred due to its fast, simple, and cost-effective technique in comparison with conventional slow freezing. However, reduced embryonic viability because of vitrification remains an unsolved problem. It has altered gamete and embryo physiology [1,2,3,4], the subcellular architecture of embryos [5,6] and gene expression [7,8,9,10,11], hence affecting developmental competence and the overall health and quality of embryos [12].

Despite the growing success of vitrification procedures, studies are still underway to examine its negative impact on cell membranes and mitochondrial bioenergetic processes triggered by oxidative stress (OS). Oxidative stress is known to impair enzyme activation, cause mitochondrial dysfunction, deplete adenosine triphosphate (ATP), affect apoptotic mechanisms, and fragment DNA, leading to developmental retardation [13,14].

In healthy cells, cytochrome c is located in the mitochondrial intermembrane spaces, where it is part of the electron transport chain. Several pro-apoptotic stimuli induce permeabilization of the mitochondrial outer membrane and promote the mobilization of cytochrome c [15]. In the intrinsic apoptotic pathway, pro-apoptotic proteins, such as Bax and Bak, translocate to the mitochondria, resulting in mitochondrial membrane permeabilization [16]. The pro-apoptotic gene, Bax, and antiapoptotic gene, Bcl2, play important roles in regulating apoptosis. The Bcl2 family is subdivided into two major groups: pro-apoptotic (Bax) and anti-apoptotic (Bcl2). Bax promotes cell death through permeabilization of the mitochondrial outer membrane in response to different cellular stresses. In contrast, Bcl2 prevents apoptosis by inhibiting the activity of Bax. This, in turn, provides a route for the release of intermembrane space proteins, as well as cytochrome c into the cytosol. Once in the cytosol, cytochrome c mediates the allosteric activation of apoptosis-protease-activating factor 1 (Apaf-1), which is required for the proteolytic maturation of caspase-9 and caspase-3. Activated caspases ultimately lead to apoptotic cell dismantling [17].

Early studies reported that the OS induced by vitrification can suppress embryonic defense capacity against reactive oxygen species (ROS) [18]. Several studies have reported that cryopreservation techniques, either slow freezing or vitrification, are associated with an increase in ROS. In the cryopreservation of two-cell murine embryos, production of hydrogen peroxide (H_2_O_2_) was observed, with slow freezing imposing a greater negative effect compared to vitrification [19]. In porcine oocytes, increases in H_2_O_2_ levels [20,21], as well as a concomitant decrease in GSH [20], following cryopreservation has been reported. This documented increase in OS associated with cryopreservation subsequently manifests as perturbations in metabolic function and gene expression patterns [22,23,24,25,26].

The high lipid contents of oocytes and embryos render them particularly susceptible to OS during cryopreservation [27,28]. The production of large amounts of ROS alters the balance between oxidation–reduction reactions and impairs the intracellular antioxidant system [29]. An imbalance in the oxidation system significantly reduces cell viability [30]. Reactive oxygen species may impact the development of embryos through cellular signaling and apoptotic regulation [31]. Cryogenic damage was also reported to greatly affect mitochondrial DNA (mtDNA) and nuclear DNA, as well as reduce cellular ATP content and other genome-related structures [29]. A safe and practical strategy of reinforcing embryos against ROS damage is, therefore, required.

The production of ROS has been reported to be counterbalanced by antioxidants, such as vitamins C and E, as well as by enzymes, such as catalase (CAT), superoxide dismutase (SOD), and glutathione peroxidase (GPx), that convert ROS to less-damaging molecules [14]. Studies reported that β-mercaptoethanol, cysteamine, cystine, cysteine, N-acetyl-L-cysteine (NAC), and SOD could be used to protect in-vitro-produced bovine embryos against OS [32]. Other studies have also reported the protective effects of antioxidants on mitochondrial activity and nuclear maturation, leading to better pre- and postimplantation embryonic development [33,34].

Glutathione (GSH) is a tripeptide (L-γ-glutamyl-L-cysteinyl-glycine) with multiple functions in living organisms [35,36]. As a carrier of an active thiol group in the form of a cysteine residue, it acts as an antioxidant either directly through interaction with reactive oxygen and nitrogen species (ROS and RNS) or indirectly through its role as a cofactor for a range of enzymes [37,38]. Several studies have reported that intracellular levels of both GSH and ROS are major determinants of preimplantation embryonic development [39,40,41]. After fertilization, GSH plays a role in the formation of the male pronucleus and is crucial for early embryogenesis up to the blastocyst stage. Previous studies have demonstrated that oocyte exposure to GSH improves postfertilization development to the blastocyst stage [42,43,44].

The supplementation of GSH in culture medium was reported to improve embryo development and quality by increasing intracellular GSH and, thereby, decreasing intracellular oxidative activity during preimplantation development [45]. In addition, GSH supplementation improved blastocyst formation and decreased the level of DNA damage by exhibiting an extensive relocation of mitochondria to the inner oocyte cytoplasm [46]. Despite reports on the benefits of GSH supplementation on preimplantation embryo development, its usefulness in improving vitrification outcomes has not been explored.

We postulate that the supplementation of culture media with GSH is effective in reducing OS, resulting in better in vitro culture (IVC) and vitrification outcomes, as observed through preimplantation development. The objectives of this study are (1) to assess the effect of GSH supplementation on the reduction of ROS generation in eight-cell embryos and (2) to determine the effect of GSH supplementation on the apoptosis- and oxidative-stress-related gene expressions of non-vitrified and vitrified preimplantation embryos.

## 2. Materials and Methods

### 2.1. Chemicals and Reagents

Pregnant mare serum gonadotropin (PMSG) and human chorionic gonadotropin (hCG) were purchased from Folligon, Intervet, Rahway, NJ, USA. Embryo manipulation tools were purchased from LabIVF (Kuala Lumpur, Malaysia). L-glutathione and other experimental reagents were purchased from Sigma Aldrich, St. Louis, MO, USA.

### 2.2. Animal Husbandry and Maintenance

Female BALB/c mice of 6–8 weeks of age were used as embryo donors. Male mice of the same strain aged 8–10 weeks were used to set up natural mating. Mice were purchased from the Laboratory Animal Facility and Management (LAFAM) Unit, Universiti Teknologi MARA. They were housed in polyurethane cages. The temperature of the holding room was maintained at 27 °C. The mice were exposed to a 12 h light:dark cycle (12:12) and provided with food pellets and water ad libitum.

### 2.3. Experimental Design

**Figure 1:**
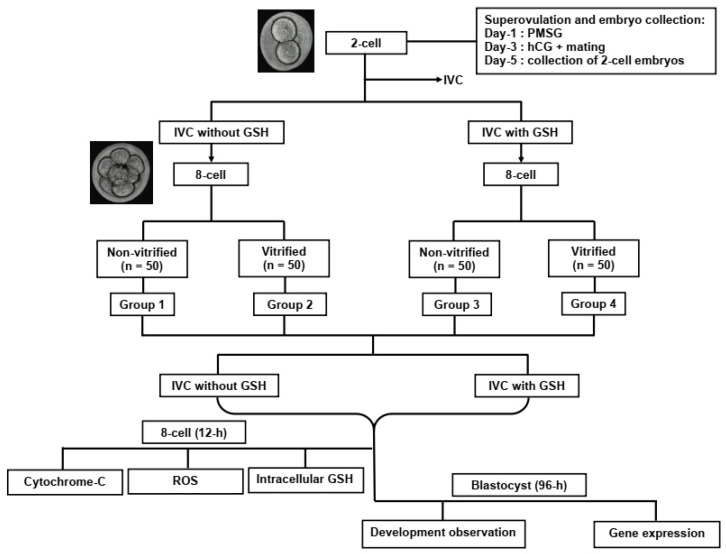
Diagrammatical representation of the study. Group 1 (GSH-free medium), Group 2 (GSH-free medium with vitrification), Group 3 (0.01 mM GSH-supplemented medium), and Group 4 (0.01 mM GSH-supplemented medium with vitrification). Abbreviation: IVC; in vitro culture.

A total of 337 embryos from female BALB/c mice were used in the experiment. Two-cell embryos were harvested from superovulated female mice after PMSG and hCG stimulation (Figure 1). The superovulated mice were then mated with fertile male mice. Female mice with copulation plugs were euthanized on day 5 after PMSG, and two-cell embryos were collected. The two-cell embryos collected were cultured either in the presence, or absence of GSH until they reached the eight-cell stage. At the eight-cell stage, embryos were randomly assigned to either non-vitrified or vitrified groups. The treatment, therefore, comprised four groups, namely Group 1 (GSH-free medium without vitrification), Group 2 (GSH-free medium with vitrification), Group 3 (0.01 mM GSH-supplemented medium without vitrification), and Group 4 (0.01 mM GSH-supplemented medium with vitrification).

After vitrification, the recovered eight-cell embryos were further cultured either in the presence or absence of GSH for another 12 or 96 h. After the 12 h culture, representative eight-cell embryos were subjected to quantification of intracellular ROS and GSH, as well as cytochrome c (Section 2.8, Section 2.9 and Section 2.10). At 96 h, the number of blastocysts was counted (development observation). After development observation was noted, the blastocysts were subjected to a gene expression study (Section 2.11). In this study, several concentrations of GSH (1.0, 0.5, and 0.01 mM) were tested. The concentration of 0.01 mM GSH was chosen, as it resulted in the best morphology and in vitro development (unpublished data).

### 2.4. Superovulation, Embryo Collection, and In Vitro Culture

Female mice were superovulated via an intraperitoneal injection of 10 IU pregnant mare serum gonadotropin (PMSG; Folligon Intervet, Rahway, NJ, USA), followed by 10 IU human chorionic gonadotropin (hCG; Folligon Intervet, Rahway, NJ, USA) 48 h later. Each female was cohabited with a single fertile male of the same strain after hCG injection.

The presence of a vaginal plug was used to confirm copulation. Mice with vaginal plugs were euthanized by cervical dislocation after 48 h of hCG injection. Embryos at the two-cell stage were flushed out of excised oviducts by the expiration of M2 medium (Sigma-Aldrich, St. Louis, MO, USA, Cat. No: M7167) through a 32-gauge hypodermic needle attached to a 1.0 mL syringe. The embryos were rinsed in M2 medium before being transferred into 50 μL droplets of either GSH-free or GSH-supplemented M16 medium (Sigma-Aldrich, St. Louis, MO, USA, Cat. No:M7292) overlaid with paraffin oil (Irvine Scientific, Santa Ana, CA, USA). They were incubated in a humidified incubator at 37 ℃ with 5% CO_2_ and 5% O_2_ until they reached the eight-cell stage. The morphological appearance and quality of the eight-cell embryos were then categorized according to Cuevas Saiz et al. [47]. Embryos in Grades I and II categories were selected to undergo culture in vitro with or without vitrification until the blastocyst stage.

### 2.5. Morphological Assessment and Grading of Two-Cell Embryos

The morphology of two-cell embryos was assessed according to Cuevas Saiz et al. [47]. Embryos were categorized as either normal or abnormal based on morphology. Normal morphology was defined as embryos having equal, rounded blastomeres absent of cell fragmentation. Meanwhile, abnormal morphology was denoted by blastomeres of unequal sizes and the presence of fragmentation. The embryos were then divided into four groups according to the experimental design (Figure 1).

### 2.6. Vitrification Warming of Eight-Cell Embryos

The vitrification protocol used in this study was adapted from Kasai et al. [48] with modifications. The eight-cell embryos were chosen based on the recommendation by Zhang et al. [49]. The eight-cell-stage embryos from IVC were exposed to EFS40 vitrification solution containing M2 medium with 40% *v*/*v* ethylene glycol, 18% *w*/*v* Ficoll 70, and 0.3 M sucrose for five minutes and then were loaded into an insemination straw and immediately plunged into liquid nitrogen (LN2). For warming, embryos were removed from LN2 and quickly exposed to a TS1 medium solution consisting of 0.75 M PBI-sucrose, 40% (*v*/*v*) ethylene glycol, 18% *w*/*v* Ficoll 70, and 0.3 M sucrose. Recovered embryos were washed in M2 medium and observed with an inverted microscope (Leica, Wetzlar, Germany: Leica DM IL LED).

### 2.7. In Vitro Culture and Preimplantation Embryo Developmental Assessment

Embryos were cultured in M16 medium with or without GSH to observe morphology and further preimplantation development. To evaluate morphological quality and preimplantation development, embryos from the two-cell to blastocyst stages were observed. Pre-compacted embryos were classified based on Cuevas Saiz et al. [47]. Embryos at two-cell, four-cell, and eight-cell stages that had equal sizes and numbers of blastomeres absent of fragmentation were defined as morphologically normal. Morula and blastocyst stages were classified based on the percentage of blastomere fragmentation: ≤10%, >10% to 20%, >25% to 35%, and >35%. Fragmentation was defined as the presence of cellular debris of blastomeric origin formed from portions of cytoplasm delimited by a cell membrane [50]. Fragmentation and normal morphology were manually scored at both times. Embryos were considered surviving if they had ≥50% cells intact immediately after warming and were considered intact and surviving if 100% of the blastomeres survived. Preimplantation development was calculated as the percentage of embryos reaching each developmental stage over the total number of embryos cultured. Observations were carried out at 24, 48, 72, and 96 h.

### 2.8. Measurement of Intracellular GSH

A total of 50 embryos at the eight-cell stage from Group 1, Group 2, Group 3, and Group 4 were observed for intracellular GSH assays. They were washed twice with 10 µM/L 4-chloromethyl-6,8-difluoro-7-hydroxycoumarin (Cell Tracker Blue CMF2HC, Molecular Probes, CA, USA). After one hour of in vitro culture, the vitrified embryos were placed in M2 medium containing 10 µm/L 4-chloromethyl-6,8-difluoro-7-hydroxycoumarin (Cell Tracker Blue CMF2HC, Molecular Probes, CA, USA) for 20 min at 37 °C. After incubation, embryos were washed in 0.1% PVA-D-PBS and mounted with antifade medium (ProLong Gold Antifading Agent; Molecular Probes, Life Technologies, California, USA). Fluorescent emissions were captured from 50 embryos using a Confocal Laser Scanning Microscope (Leica CLSM, Wetzlar, Germany) with a UV filter. Throughout the experiment, the embryos were manipulated under low light to minimize environmental influences. The fluorescent intensities of the images were quantified using LAS AF Lite version 2.6 by mean gray values of fluorescence. Background fluorescent values were subtracted from the final values before analyzing the statistical difference among the groups.

### 2.9. Measurement of Intracellular ROS

A total of 50 embryos at the eight-cell stage from Group 1, Group 2, Group 3, and Group 4 were used to measure the intracellular ROS levels using 2′, 7′-dichlorofluoresceindiacetate fluorescence assays. Embryos were fixed with 4% paraformaldehyde overnight as previously described [51]. The embryos were washed twice with phosphate-buffered saline (PBS) and then incubated for 30 min in 1 mL of 10 µM 2′, 7′-dichlorofluorescein diacetate fluorescent probe (DCHFDA, Molecular Probes, Life Technologies, CA, USA) at 37 °C. After washing with 1% PBS-BSA, embryos were mounted with antifade medium (ProLong Gold Antifading Agent; Molecular Probes, Life Technologies, CA, USA). Fluorescent emissions from the embryos were acquired using a Confocal Laser Scanning Microscope (Leica CLSM) with a UV filter at 460 nm to quantify the fluorescent signal intensities (pixels). The fluorescent intensities of images were quantified using LAS AF Lite version 2.6 (Leica Microsystem CMS GmbH, Wetzlar, Germany) by mean gray values of fluorescence. Background fluorescent values were subtracted from the final values before analyzing the statistical difference among the groups.

### 2.10. Measurement of Cytochrome C Activity

The activity of cytochrome c in the eight-cell stage (*n* = 50 in each group) was assessed using fluorescent probes according to the manufacturer’s instructions. The embryos were fixed with 4% paraformaldehyde overnight, permeabilized with 0.1% Triton X-100 (Sigma Aldrich, St. Louis, MO, USA) for 10 min, and blocked with 1% BSA (Sigma Aldrich, St. Louis, MO, USA) for one hour at room temperature. The embryos were then incubated with primary antibody, which was a cytochrome c monoclonal antibody (Thermo Fisher Scientific), at 2 µg/mL in 0.1% BSA, incubated for three hours at room temperature, and then labelled with goat anti-mouse IgG (H + L) Superclonal alexa fluor 488 conjugate as a secondary antibody (Thermo Fisher Scientific) at a dilution of 1:2000 for 45 min at room temperature. Images were captured at 40X magnification using a Confocal Laser Scanning Microscope (Leica, CLSM), and the quantification of fluorescent intensities was carried out using image analysis software (LAS AF Lite version 2.6, Leica Microsystem CMS GmbH, Wetzlar, Germany) by mean gray values of fluorescence. Background fluorescent values were subtracted from the final values before analyzing the statistical difference among the groups.

### 2.11. Reverse Transcription and Microfluidic qPCR

The quantification of apoptosis-inducing factors (*Bax* and *Bcl2*) and oxidative-stress-related genes (*Gpx1* and *Sod1*) in embryos subjected and not subjected to vitrification procedures was carried out with a BioMark HD system (Fluidigm Microfluidic Technology, San Francisco, CA, USA). In this study, blastocysts obtained from in vitro culture of non-vitrified and vitrified groups were used. Preliminary screening was performed to obtain an adequate RNA concentration of A_260_/A _280_ (1.8–2.0) from 30 blastocysts for each biological replicate. A total of 120 blastocysts were used as an RNA source in the gene expression analysis. RT-qPCR Kits (Macherey-Nagel, Düren Germany, Cat. No.740902.50) were used. Complementary DNA (cDNA) synthesis was performed using Reverse Transcription Master Mix from Fluidigm^®^ according to the manufacturer’s protocol with random primers in a final volume of 5 µL containing 6 ng of total RNA. The cDNA samples were diluted by adding 20 µL of low-TE buffer (10 mM Tris; 0.1 mM EDTA; pH = 8.0 (TEKNOVA)) and stored at −20 °C. A total of 1.25 µL of each diluted cDNA was used for multiplex pre-amplification with Fluidigm^®^ PreAmp Master Mix at 19 cycles. An amount of 1 µL of pre-amplification target genes related to the apoptosis pathway was contained in a total volume of 5 µL reaction sample. Data were normalized to the expression level of the reference gene (*Gapdh*) in each sample. The primer sequences are listed in Table 1. The cycle threshold (Ct) value was defined as the number of PCR cycles in which the fluorescence signal exceeded the detection threshold value. The 2−ΔΔCq method was used to calculate the fold change in the expression [52]. The control was set to 100%, and the experimental and control samples were compared [53].

### 2.12. Statistical Analyses

Statistical analyses were performed using SPSS software for Windows, version 23 (Statistical Package for Social Sciences, Armonk, NY, USA). Graphs were illustrated using GraphPad Prism 8 for Windows, version 8.0.1 (GraphPad Software., La Jolla CA, USA). Two-cell embryos and blastocysts were scored based on qualitative assessment. Statistical differences in morphology among groups were determined using the chi-squared test, with significance determined at *p* < 0.05. Fluorescence intensity in the ROS analysis was determined using one-way ANOVA, followed by Tukey’s multiple comparison tests. Data were expressed as means ± standard error for the mean (SEM). For gene expression analysis, values for delta-delta CT (ΔΔCT) were obtained from Fluidigm Real-time PCR analysis software. All the results were statistically significant at a level of *p* < 0.05.

## 3. Results and Discussion

### 3.1. Exogenous GSH Supplementation Improved Preimplantation Embryo Morphology

The most widely used method of embryo selection is the visual assessment of embryo morphology. Numerous parameters can be evaluated at various developmental stages, providing valuable information about embryonic quality [54]. Embryos can be graded based on the morphology of their pronuclei on day 1 after fertilization [55,56], the number and shape of blastomeres and the degree of fragmentation on days 2 or 3 [13,57,58], or the morphology of the blastocyst on day 5 [24,59].

**Figure 2:**
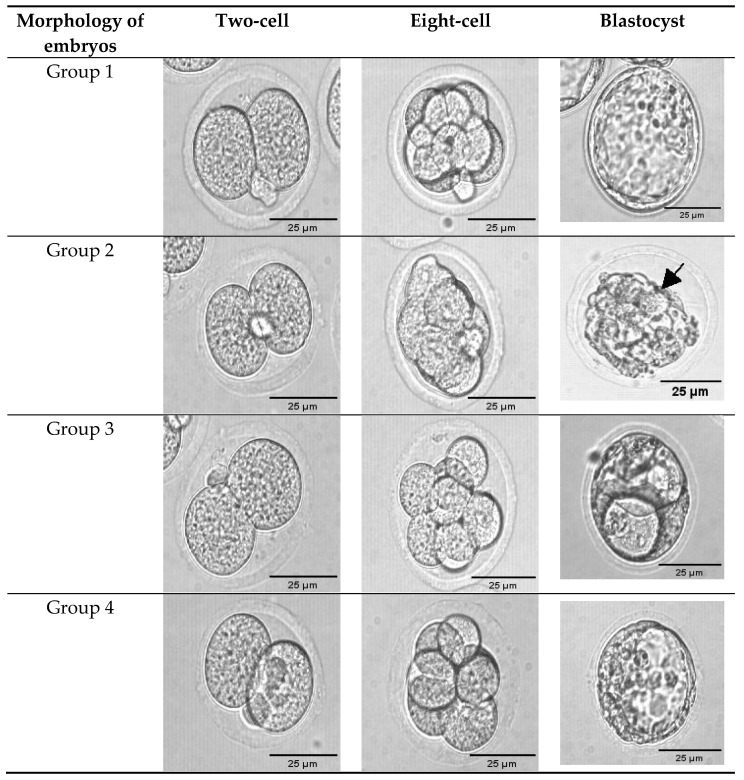
Morphologies of two-cell in vitro cultures to eight-cell embryos that underwent vitrification and without vitrification were observed until development to blastocyst stage from Groups 1–4. Arrows show fragmented embryos. Group 1 (GSH-free medium); Group 2 (GSH-free medium with vitrification); Group 3 (0.01 mM GSH-supplemented medium), Group 4 (0.01 mM GSH-supplemented medium with vitrification). The scale bar for the images represents 25 µm.

Images of embryos with or without GSH treatment were morphologically scored using the scoring system of Cuevas Saiz et al. [47]. Each embryo was quantitatively classified according to the size of the blastomere and embryonic fragmentation, as shown in Figure 2. The degree of fragmentation was initially described as the embryonic volume occupied by anucleate cytoplasmic fragments and is given as a percentage [60]. Glutathione supplementation was seen to significantly increase (*p* < 0.05) the number of Grade-1 blastocysts from 69% to 80% (Groups 1 and 3, respectively). Similarly, GSH supplementation also significantly increased postvitrification blastocyst formation from 63% to 81% (Groups 2 and 4, respectively) (Table 2). Similar to our findings, prior research has found that an increasing proportion of oocytes develop in GSH-supplemented maturation medium after IVF reaches the blastocyst stage [61,62].

It has been reported that a correlation exists between embryo morphology and viability [63,64]. The size and shape of blastomeres, the presence of extruded cells or fragmentation, compaction, the color of embryos, and the developmental stage reached at a particular point after fertilization are all significant parameters in embryo morphology assessment [65]. A previous study showed a correlation between oocyte cytoplasmic morphology and ICSI-induced fertilization rate and embryo quality. Significantly lower fertilization, embryo cleavage, and embryo quality have been observed in oocytes with cytoplasmic inclusions compared to oocytes with normal cytoplasm [66,67]. Fragmentation has also been linked to an increased risk of chromosomal abnormalities [68,69], leading to reduced blastocyst formation and implantation rates [70]. Viability is determined by embryo quality. Rapidly dividing embryos are more viable and have greater developmental potential than slowly dividing embryos [71,72].

### 3.2. Exogenous GSH Supplementation Enhanced Preimplantation Embryo Development

The effect of GSH supplementation on embryo development is shown in Table 2. The addition of GSH resulted in significantly higher numbers of embryos reaching the blastocyst stage. The number of blastocysts was significantly higher in Group 1 (GSH-free medium) compared to Group 3 (GSH-supplemented medium) (69% vs. 80% respectively). A previous study also demonstrated that embryos cultured in GSH-supplemented medium showed better development compared to embryos cultured in GSH-free medium [73]. In addition, oocyte exposure to GSH has improved postfertilization development to the blastocyst stage [27,46,73,74,75]. Supplementation of GSH in culture medium was reported to improve embryo development and quality by increasing intracellular GSH and decreasing intracellular oxidative activity during preimplantation development [75]. Another study reported that GSH improved blastocyst formation and decreased DNA damage by exhibiting the extensive relocation of mitochondria to the inner oocyte cytoplasm [46].

Our findings showed that GSH improved blastocyst development of vitrified embryos from 63.0% (Group 2) to 81.0% (Group 4) (*p* < 0.05) (Table 2). As discussed earlier, most studies have investigated the impact of GSH supplementation on development in vitro. There have been no published data on the impact of GSH supplementation on the cryotolerance of vitrified embryos. Further research on the possible mechanism of GSH-enhanced cryotolerance in vitrified embryos is, therefore, warranted.

### 3.3. Exogenous GSH Supplementation Reduced Oxidative Stress in Vitrified Preimplantation Embryos

**Figure 3:**
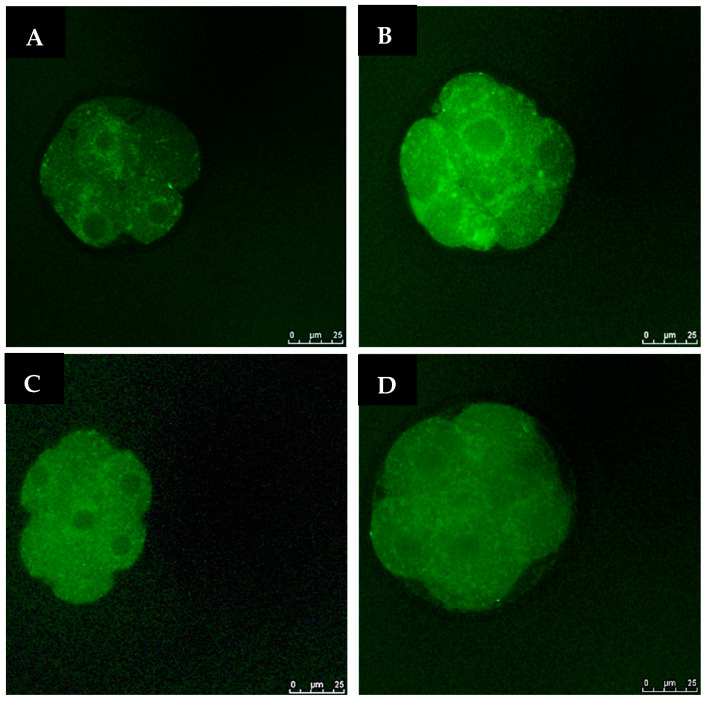
Fluorescent photomicrograph of embryos showing intracellular ROS contents. Images are viewed under 400× magnification. (**A**): Group 1 (GSH-free medium); (**B**): Group 2 (GSH-free medium with vitrification); (**C**): Group 3 (0.01 mM GSH-supplemented medium), (**D**): Group 4 (0.01 mM GSH-supplemented medium with vitrification). The scale bar for the images represents 25 µm.

**Figure 4:**
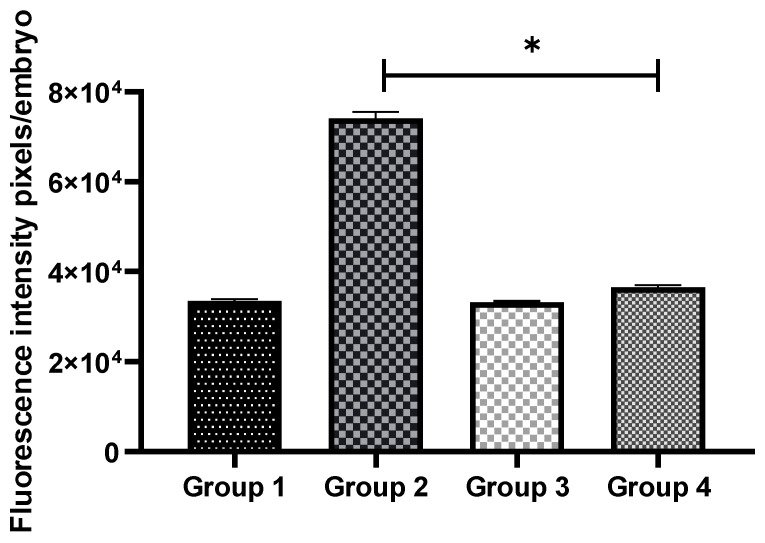
Comparison of intracellular ROS levels in non-vitrified and vitrified embryos. Data are expressed as the means ± SEM. * indicates *p* < 0.05. Data were analyzed using one-way ANOVA with post hoc Tukey multiple comparison tests. Group 1 (GSH-free medium); Group 2 (GSH-free medium with vitrification); Group 3 (0.01 mM GSH-supplemented medium), Group 4 (0.01 mM GSH-supplemented medium with vitrification).

The intracellular ROS levels were examined to investigate whether OS could be reduced by GSH supplementation. The intensities of fluorescence generated by oxidized DCHFDA in eight-cell embryos are shown for all the groups (Figure 3). In non-vitrified groups (Groups 1 and 3), there was no significant difference in embryonic OS levels. However, in vitrified groups (Groups 2 and 4), GSH treatment significantly reduced ROS levels (*p* < 0.05) (Figure 4).

Previous studies have shown that elevated levels of ROS induced by vitrification are a possible cause of low preimplantation development rates [76,77,78]. In mammalian oocytes, vitrification has been reported to damage the endogenous antioxidant system [14] and mitochondria [79]. Mitochondria contain multiple electron carriers that can produce ROS. Damage to the organelle can result in an imbalance between the production and scavenging of ROS, resulting in net ROS production. As a result, ROS is partially responsible for the apoptosis or developmental arrest in embryos associated with cryopreservation. Under normal conditions, embryos have a defense mechanism against ROS in the form of antioxidant enzymes, including SOD and CAT. However, there is a possibility that embryo freezing disrupts these protective mechanisms [6].

An increase in ROS production in mammalian embryos has been shown to cause developmental arrests [80], embryonic fragmentation, or programmed cell death [45,81]. ROS-induced programmed cell death in embryos was attributed to changes in mitochondrial function, leading to higher production of ROS [82]. The addition of antioxidants was reported to prevent embryonic OS and apoptosis [83]. Therefore, we proposed that supplementation of GSH during vitrification was able to reduce OS by regenerating the GSH balance in an embryo. Glutathione supplementation prevents lipid peroxidation by removing excessive ROS in media [84]. It was reported that GSH improved oocyte fertilization by reducing the number of disulfide bonds in the zona pellucida, rendering them less rigid [27].

### 3.4. Exogenous GSH Supplementation Increased Endogenous GSH Levels in Vitrified Preimplantation Embryos

**Figure 5:**
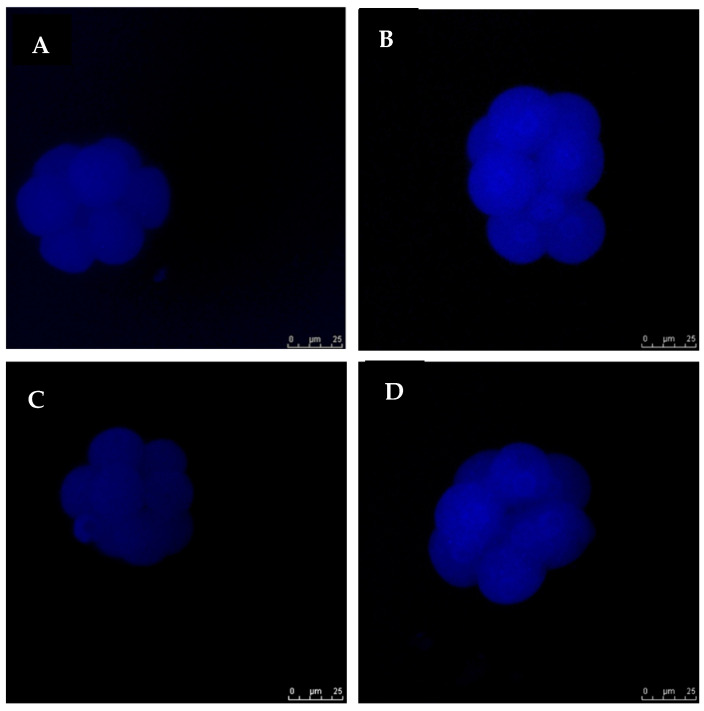
Fluorescent photomicrograph of embryos showing intracellular GSH contents. Images are viewed under 40× magnification. (**A**): Embryo from Group 1 (GSH-free medium); (**B**): embryo from Group 2 (GSH-supplemented medium); (**C**): embryo from Group 3 (GSH-free medium with vitrification); (**D**): embryo from Group 4 (GSH-supplemented medium with vitrification).

**Figure 6:**
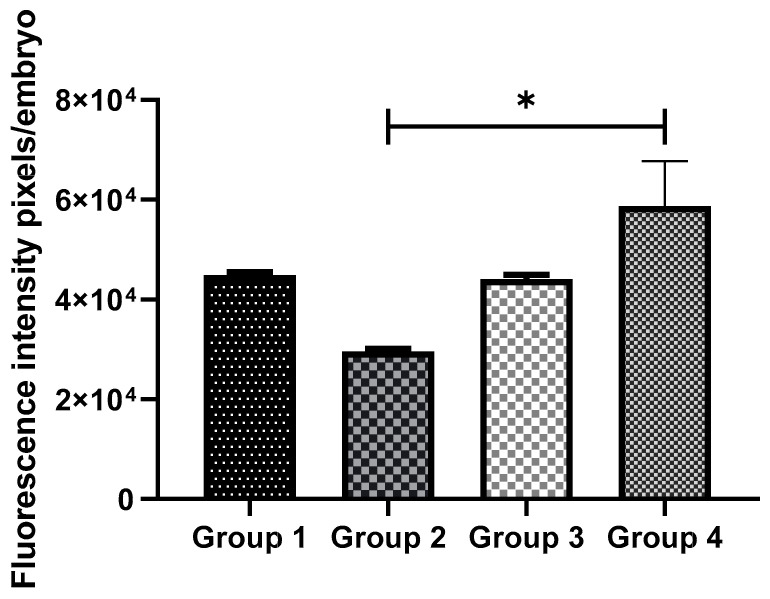
Comparison of intracellular GSH levels between non-vitrified and vitrified embryos. Data are expressed as significant differences between the means ± SEM. * indicates *p* < 0.05. Group 1 (GSH-free medium); Group 2 (GSH-free medium with vitrification); Group 3 (0.01 mM GSH-supplemented medium); Group 4 (0.01 mM GSH-supplemented medium with vitrification).

The intracellular levels of GSH in eight-cell embryos from all the groups were examined. Figure 5 shows images of embryos stained with 4-chloromethyl-6,8-difluoro-7-hydroxycoumarin. No remarkable difference in intracellular GSH levels was noted between non-vitrified embryos in Groups 1 and 2. However, the intracellular GSH level in vitrified embryos was significantly higher (*p* < 0.05) in Group 4 compared to Group 3 (Figure 6). The addition of GSH into culture media was, therefore, able to reduce ROS levels and increase the viability of vitrified embryos.

Exogenous GSH supplementation during in vitro culture has been reported to act as a defense mechanism against ROS by improving the intracellular GSH system [75,84]. An earlier study stated that the supplementation of 1 mM GSH to maturation medium stimulated intracellular GSH and maintained the redox balance in bovine IVF embryos [75]. However, further research is required to determine the efficiency in which specific embryonic stages acquire and use exogenous GSH from the culture medium. It is known that endogenous GSH in an embryo is essential for its protection against OS and other forms of cellular injury [84,85]. The degradation of GSH occurs exclusively in the extracellular space and on the surfaces of cells that express γ-glutamyl transpeptidase (GGT). The enzyme may transfer the γ-glutamyl moiety of GSH to amino acids and peptides. Frequently, cells take up the products of GSH hydrolysis as individual amino acids or as dipeptides. The balance between GSH production, consumption, and transportation determines the intra- and extracellular GSH levels [36]. The molecular mechanism by which GSH protects embryos from damage inflicted by vitrification, however, remains to be elucidated.

### 3.5. Exogenous GSH Supplementation Decreased Cytochrome C Expression in Vitrified Preimplantation Embryos

**Figure 7:**
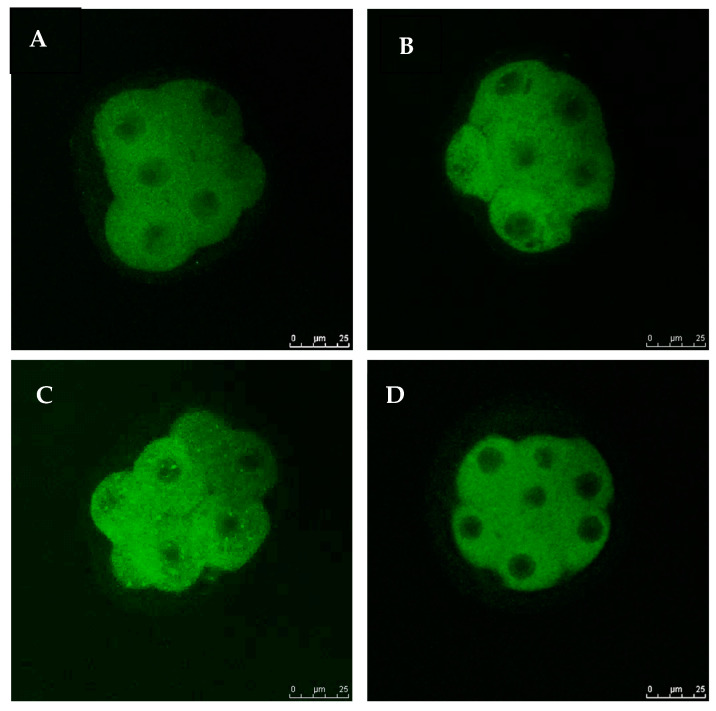
Fluorescent photomicrograph of embryos showing cytochrome c expression. Images are viewed under 40X magnification. (**A**): Embryo from Group 1 (GSH-free medium); (**B**): embryo from Group 2 (GSH-supplemented medium); (**C**): embryo from Group 3 (GSH-free medium with vitrification); (**D**): embryo from Group 4 (GSH-supplemented medium with vitrification).

**Figure 8:**
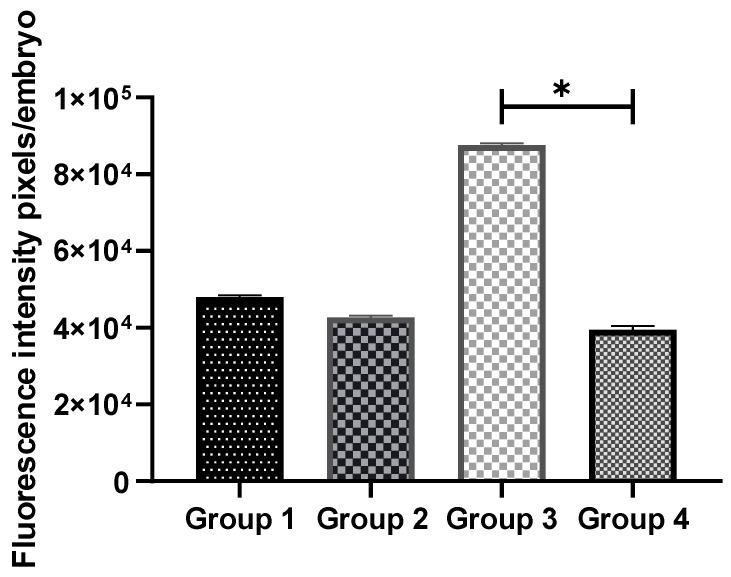
Comparison of cytochrome c expression between non-vitrified and vitrified embryos. Data are expressed as significant differences between the means ± SEM. * indicates *p* < 0.05. Group 1 (GSH-free medium); Group 2 (GSH-supplemented medium); Group 3 (GSH-free medium with vitrification); Group 4 (GSH-supplemented medium with vitrification).

The expression of cytochrome c in non-vitrified and vitrified eight-cell stage embryos cultured with and without 0.01 mM GSH supplementation was analyzed using immunocytochemical staining (Figure 7). No significant difference in cytochrome c expression was observed between the non-vitrified embryos in Groups 1 and 2. However, 0.01 mM GSH-supplemented medium (Group 4) exhibited a significantly lower (*p* < 0.05) expression of cytochrome c compared to Group 3 (Figure 8).

The processes of vitrification and thawing have been reported to damage the endogenous antioxidant system [21] and mitochondrial distribution in mouse embryos and to effect embryonic development [86].

Mitochondria are organelles for ATP production that are important for controlling cell growth, dynamic response, signaling, and apoptosis in mammalian cells. Damage caused by excessive ROS results in the release of cytochrome c into the cytoplasm, which is one of the messengers of mitochondria-dependent apoptotic pathways [44]. Oxidative stress causes the release of cytochrome c and other apoptogenic factors from mitochondria, which then activates apoptosis [87].

In this study, 0.01 mM GSH supplementation in the culture medium significantly decreased the expression of cytochrome c in vitrified eight-cell embryos, suggesting that GSH supplementation improved mitochondrial function and cryotolerance. Similar to our results, previous studies reported that the addition of GSH to mouse embryo culture medium reduced ROS in embryos and increased the percentage of blastocysts [88]. Similar results were obtained in porcine [42] and bovine [75] models.

Therefore, we concluded that the addition of GSH to the culture medium could suppress ROS in the culture medium and reduce the amount of ROS transported into embryos. Thus, exogenous GSH supplementation could serve as a defense against ROS during vitrification through the maintenance of intracellular GSH.

### 3.6. Exogenous GSH Supplementation Upregulated the Expression of Bcl2 Gene and Downregulated the Expression of Bax Gene in Vitrified Preimplantation Embryo

**Figure 9:**
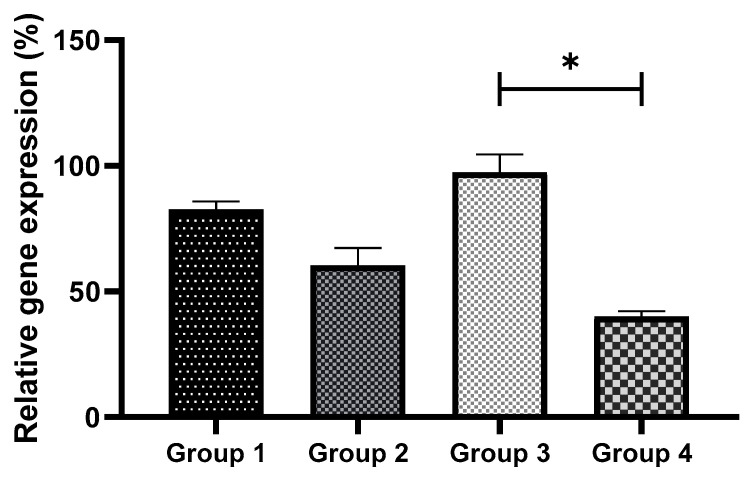
The effect of GSH supplementation on pro-apoptotic *Bax* gene expression in non-vitrified and vitrified groups. Data are expressed as the means ± SEM. * *p* < 0.05. Group 1 (GSH-free medium); Group 2 (GSH-supplemented medium); Group 3 (GSH-free medium with vitrification); Group 4 (GSH-supplemented medium with vitrification).

**Figure 10:**
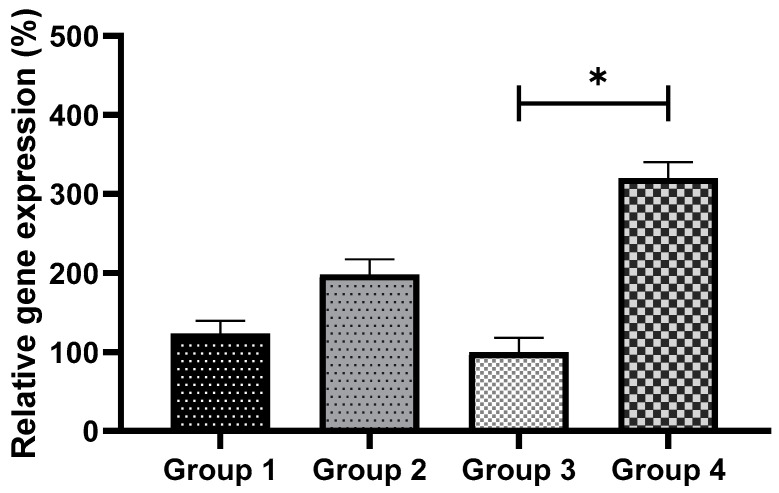
The effect of GSH supplementation on *Bcl2* gene expression in non-vitrified and vitrified groups. Data are expressed as the means ± SEM. * *p* < 0.05. Group 1 (GSH-free medium); Group 2 (GSH-supplemented medium); Group 3 (GSH-free medium with vitrification); Group 4 (GSH-supplemented medium with vitrification).

The expressions of *Bax* and *Bcl2* (apoptosis-related genes) were analyzed for all the groups, as shown in Figure 9; Figure 10, respectively. No significant differences were observed in the expression of *Bax* across the non-vitrified groups. However, in the vitrified groups, GSH supplementation (Group 3) showed a significantly lower (*p* < 0.05) *Bax* expression compared to Group 4 (Figure 9). In tandem, *Bcl2* expression was found to be significantly higher (*p* < 0.05) in Group 4 compared to Group 3 (Figure 10). The results from the *Bax* and *Bcl2* expressions suggest that GSH supplementation reduced apoptosis in vitrified embryos.

The Bcl2 family has been shown to regulate mitochondrial outer membrane permeabilization and can be either pro-apoptotic (genes such as *Bax*) or anti-apoptotic (genes such as *Bcl2*) in the mitochondrion-mediated apoptosis pathway [21]. Anti-apoptotic genes, such as *Bcl2*, prevent apoptosis by inhibiting the release of cytochrome c from mitochondria. It has been shown that vitrification increases the mRNA levels of *Bax* in mouse oocytes [89] and bovine oocytes [90].

In our study, we measured the expressions of apoptosis-related genes via RT-qPCR, demonstrating that vitrification significantly increased the *Bax* mRNA level, contributing to the induction of apoptosis in vitrified mouse embryos, in agreement with reports in bovine [91,92], porcine [93], murine, and oocyte [94] studies. In addition, our results showed that GSH supplementation significantly decreased the *Bax* mRNA level and increased the *Bcl2* mRNA level, illustrating that GSH supplementation in culture medium inhibited mitochondrion-mediated apoptosis by regulating the expression of the pro- (*Bax*) and anti-apoptotic (*Bcl2*) genes of the Bcl2 family, which indicated a high developmental rate of the embryos (Table 2). These results are consistent with previous research by Circu and Aw [95], which showed that supplementing with GSH upregulated the anti-apoptotic *Bcl2* gene, increasing the levels of GSH in cells and indicating rapid embryonic development.

This finding was supported by the cytochrome c expression results, which showed that culture medium supplemented with 0.01 mM GSH had a significantly lower cytochrome c level and that reduced apoptosis resulted in a greater number of Grade-1 embryos and improved preimplantation development.

### 3.7. Exogenous GSH Supplementation Upregulated the Expression of Gpx1 and Sod1 Genes in Vitrified Preimplantation Embryo

**Figure 11:**
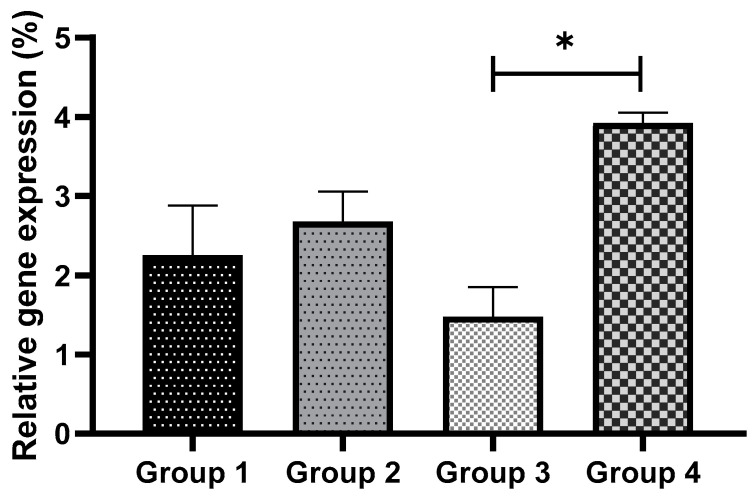
The effect of GSH supplementation on *Gpx1* gene expression in non-vitrified and vitrified groups. Data are expressed as the means ± SEM. * indicates *p* < 0.05. Group 1 (GSH-free medium); Group 2 (GSH-supplemented medium); Group 3 (GSH-free medium with vitrification); Group 4 (GSH-supplemented medium with vitrification).

**Figure 12:**
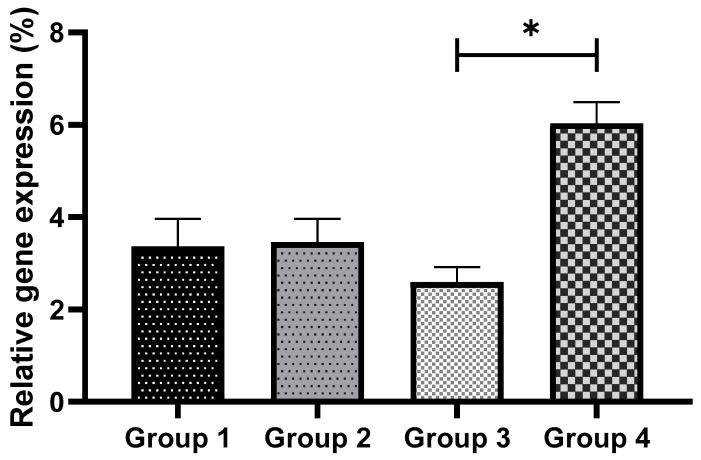
The effect of GSH supplementation on *Sod1* gene expression in non-vitrified and vitrified groups. Data are expressed as the means ± SEM. * *p* < 0.05. Group 1 (GSH-free medium); Group 2 (GSH-supplemented medium); Group 3 (GSH-free medium with vitrification); Group 4 (GSH-supplemented medium with vitrification).

The relative transcript abundances of glutathione peroxidase 1 (*Gpx1*) and superoxide dismutase 1 (*Sod1*) produced in response to GSH supplementation in culture medium with and without vitrification are shown in Figure 11 and Figure 12, respectively. There were no significant differences in the expression levels of *Gpx1* and *Sod1* in non-vitrified groups (Group 1 vs Group 2). In contrast, 0.01 mM GSH supplementation in the culture medium resulted in a significant (*p* < 0.05) upregulation of *Sod1* in vitrified preimplantation embryos (Group 3 vs Group 4) (Figure 12). Similarly, a previous study reported that *Sod1* gene expression was associated with the superior quality of in-vitro-produced murine embryos [96].

With regard to relative *Gpx1* transcript abundance in vitrified preimplantation embryos, a significant (*p* < 0.05) decrease in *Gpx1* expression was observed in Group 3 embryos. The present study confirms a previous report that cryopreservation decreased antioxidant enzyme expression in murine two-cell embryos [97]. Cryopreservation induced the downregulation of *Gpx1* and *Sod1* expressions, indicating that cryopreservation may alter the gene expressions of oocytes and embryos [98].

The *Gpx1*-encoded glutathione peroxidase-1 protein is essential for apoptosis. It helps in hydrogen peroxide catabolism and cellular redox balance. Glutathione peroxidase *(GPx*) is essential for the equilibrium between the two-step enzymatic antioxidant reaction that prevents damage to tissues and cells, which involves the transfer of SOD from superoxide anion to hydrogen peroxide, as well as *GPx* from hydrogen peroxide to water [99]. Glutathione peroxidase 1 plays the primary protective role in managing oxidative injury and death from ROS, as indicated by evidence derived from *Gpx1* knockout mice [100]. In accordance with our findings, GSH supplementation in vitrified embryos (Group 4) resulted in significant (*p* < 0.05) increases in the expressions of *Gpx1* and *Sod1*.

Therefore, these studies demonstrated the importance of the *Gpx1* and *Sod1* genes in preimplantation embryo cryopreservation. GSH is the predominant antioxidant in all cell types [101], and it is not surprising that the glutathione cycle is required for freeze–thaw tolerance. These studies proved the significance of exogenous GSH in reducing freeze–thaw stress and improving cryotolerance. However, the exact mechanism by which the supplementation of GSH to the culture medium increased the quality of the vitrified embryos is still unknown. Based on the present results, the GSH-mediated increase in *Sod1* and *Gpx1* transcript abundances is suggested to help in the balance and maintenance of cellular redox. This would result in improved embryonic cryotolerance.

## 4. Conclusions

In conclusion, the supplementation of 0.01 mM L-glutathione (GSH) in culture media increased intracellular GSH levels, as well as reduced mitochondrial dysfunction, ROS levels, and apoptosis. These mechanisms may contribute to improved preimplantation development in both non-vitrified and vitrified embryos. Our findings cast a new light on the novel use of GSH as a simple yet effective means to improve vitrification outcomes.

## Figures and Tables

**Table 1:** Primer sequences of the target genes used for quantitative reverse transcription polymerase chain reaction.

Gene	Primer Sequence (5′–3′) Forward	Primer Sequence (3′–5′) Reverse	Accession Number
*Gapdh*	CAAGGTCATCCCAGAGCTGAA	CAGATCCACGACGGACACA	NM_001289726.N
*Bax*	ATGTGTGTGGAGAGCGTCAA	CTGATCAGCTCGGGCACTTTA	NM_007527.N
*Bcl2*	GCGTGGTTGCCCTCTTCTA	GATGCCGGTTCAGGTACTCA	NM_009741.N
*Gpx1*	TCGGTTTCCCGTGCAATCA	GTCGGACGTACTTGAGGGAA	NM_008160.N
*Sod1*	CTCACTCTCAGGAGAGCATTCC	TTCCACCTTTGCCCAAGTCA	NM_011434.N

Abbreviations: *Gapdh*, glyceraldehyde-3-phosphate dehydrogenase; *Bax, Bcl2*-associated X apoptosis regulator; *Gpx1*, glutathione peroxidase 1; *Sod1*, superoxide dismutase 1.

**Table 2:** Effect of GSH supplementation on the development of blastocysts.

Groups	No. of Two-Cell Embryos (%)	No. of Eight-Cell Embryos (%)	No. of Blastocysts (%)
	*n*	(*n* ± SD)	(*n* ± SD)
Group 1	76 (100%)	21.00 ± 3.49 (83%)	17.67 ± 3.06 (69%) ^a^
Group 2	60 (100%)	15.00 ± 2.86 (75%)	12.67 ± 1.89 (63%) ^a^
Group 3	111 (100%)	31.67± 5.14 (86%)	29.67 ± 0.62 (80%) ^b^
Group 4	90 (100%)	26.67± 2.78 (89%)	24.33 ± 1.43 (81%) ^c^

Each categorical variable is described as a percentage, with between-group differences tested by chi-squared test (*p* < 0.05). Different superscripts within the same column (no. of blastocysts) represent significant differences (*p* < 0.05).

## Data Availability

All data is contained within the article.
